# Long Term Outcome of Severe Anaemia in Malawian Children

**DOI:** 10.1371/journal.pone.0002903

**Published:** 2008-08-06

**Authors:** Kamija S. Phiri, Job C. J. Calis, Brian Faragher, Ernest Nkhoma, Kondwani Ng'oma, Bridget Mangochi, Malcolm E. Molyneux, Michaël Boele van Hensbroek

**Affiliations:** 1 Malawi-Liverpool-Wellcome Trust Clinical Research Programme, College of Medicine, Blantyre, Malawi; 2 Emma Children's Hospital AMC, University of Amsterdam, Amsterdam, the Netherlands; 3 Liverpool School of Tropical Medicine, Liverpool, United Kingdom; University of Bern, Switzerland

## Abstract

**Background:**

Severe anaemia is a common, frequently fatal, condition in African children admitted to hospital, but its long term outcome is unknown. Early reports that survivors may be at risk of additional late morbidity and mortality may have significant implications for child survival in Africa. We assessed the short and long term outcome of severe anaemia in Malawian children and identified potential risk factors for death and further severe anaemia.

**Methodology and Findings:**

For 18 months, we followed up children (6–60 months old) presenting to hospital with severe anaemia (haemoglobin ≤5g/dl) and their hospital and community controls with the aim to compare all cause mortality and severe anaemia recurrence rates between the groups, and to identify risk factors for these adverse outcomes. A total of 377 cases, 377 hospital controls and 380 community controls were recruited. Among cases, the in-hospital mortality was 6.4% and post-discharge all cause mortality was 12.6%, which was significantly greater than in hospital controls (2.9%) or community controls (1.4%) (Log rank test, p<0.001). The incidence of recurrence of severe anaemia among the cases was 0.102 per child-year (95% Confidence Interval 0.075–0.138), and was significantly higher than the 0.007 per child-year (95% CI 0.003–0.015) in the combined controls (p<0.0001). HIV was the most important risk factor both for post-discharge mortality (Hazard Ratio 10.5, 95% CI 4.0–27.2) and for recurrence of severe anaemia (HR 5.6, 95% CI 1.6–20.1).

**Conclusions:**

Severe anaemia carries a high ‘hidden’ morbidity and mortality occurring in the months after initial diagnosis and treatment. Because severe anaemia is very common, this is likely to contribute importantly to overall under-five mortality. If not adequately addressed, severe anaemia may be an obstacle to achievement of the Millennium development goal No.4 on child survival. Strategies to diagnose and properly treat HIV infected children early most likely will reduce the high post-discharge mortality in severe anaemia.

## Introduction

Severe anaemia is a common condition causing significant morbidity and mortality in children in Africa. Of children admitted to hospital, 12–29% have severe anaemia requiring a blood transfusion and the in-hospital mortality is commonly very high, ranging between 4% and 10% [1], [2], [3]. Although the condition may be caused by various aetiological factors, most important being nutritional deficiencies and infections such as HIV and malaria [2], [4], [5], these factors usually occur simultaneously and are complexly related. In these parts of the world, hospitals are commonly challenged to manage severely anaemic children with poor or absent diagnostic facilities.

Investigators in Kenya reported unexpectedly high post-discharge mortality and recurrent severe anaemia rates in children within two months of a severe anaemia episode [2]. There has been no subsequent attempt to confirm or investigate these findings. An excess risk of death in this large group could contribute an important, and potentially correctable, component to the high mortality rate among young children in Africa.

In this study our first aim was to compare rates of survival and of recurrence of severe anaemia over 18 months between children admitted to hospital with severe anaemia, children admitted to hospital for other reasons and healthy children in the local community. Secondly we aimed to investigate risk factors for recurrent severe anaemia and all cause mortality during an 18-month follow-up period.

## Methods

This study was the longitudinal part of a case-control study investigating the aetiological factors for severe anaemia in children [6]. We followed up three groups of children namely: (1) those who were admitted to hospital for treatment of severe anaemia (cases), (2) those who presented to hospital for other causes (hospital controls) and (3) those from the community of each case (community controls). It was conducted in southern Malawi at Queen Elizabeth Central hospital in Blantyre and Chikwawa District hospital. In Blantyre (urban site) malaria is seasonal, while in Chikwawa (rural site) malaria transmission is intense throughout the year.

Children were recruited from July 2002 to July 2004 and followed up until February 2006 from the hospital's Paediatric Accident and Emergency Unit (Blantyre) or Under-fives Clinic (Chikwawa). Each of these is a facility available to the public without charge, functioning in daylight hours for six days each week. Severely anaemic children (*cases*) were recruited if they had a blood haemoglobin concentration ([Hb]) of less than 5.0 g/dl, were aged 6–60 months and had not received a blood transfusion during the preceding four weeks. A child was not enrolled if the anaemia was known to be due to malignancy or trauma. For each *case* a Hospital Control (*HC*) was recruited the following day on presentation to the same out-patient department for a condition other than severe anaemia, and when the index *case* was discharged from hospital a Community Control (*CC*) was recruited from among apparently healthy children in the community. The guardian of the index case assisted in identifying the nearest child not part of the immediate family residing within 100–1000metres of the home of the index case. *HC* and *CC* had to be aged between 6 and 60 months, and not severely anaemic. Written informed consent was obtained from the guardians of the children and the study was approved by the ethics committees of the University of Malawi and the Liverpool School of Tropical Medicine, UK.

### Clinical procedures

The process of recruitment included a detailed medical history and physical examination. Samples of venous blood, urine and stool were collected. Children requiring hospitalisation (all cases and a minority of *HC*s) received 24 hour care in a specialised research ward while in hospital. An array of laboratory tests to determine the aetiology of the clinical condition (described below) were performed [6]. *Cases* received an urgent blood transfusion (20mls whole blood per kilogram body weight). Severe malaria was treated with parenteral quinine until the child was able to take oral medication, then sulfadoxine-pyrimethamine was given according to national policy at that time [7]. Antibiotics were given only when clinically indicated. All correctable conditions were treated according to a standard protocol before children were discharged from hospital.

### Participant follow-up

All three study groups (*cases*, *HC* and *CC*) were actively followed up at 1, 3, 6, 12 and 18 months from date of discharge. Guardians were additionally asked to return with the child to a study clinic whenever the child was sick. During each follow-up visit, a standard clinical form was completed and, if necessary, the child was treated by the attending clinician. At the time of this study, anti-retroviral therapy (ART) was not available to children in Malawi. Deaths and severe anaemia episodes were recorded, and if they occurred outside the study clinics, they were investigated as completely as possible using the patient's ‘Health Passport’ book, hospital records and home interviews with parents or guardians. Deaths occurring during the initial hospitalisation period were recorded as in-hospital mortality. Deaths occurring after discharge but during the study follow-up period were recorded as post-discharge all cause mortality and information surrounding the death was collected using a verbal autopsy [8].

### Laboratory methods

All laboratory assays were done in all study groups, unless otherwise stated and were blinded to patient study group where possible. Haemoglobin concentration was measured on site using the HemoCue B-Haemoglobin analyser (HemoCue, Ängelholm, Sweden) and subsequently by Coulter counter analyser (Beckman Coulter, Durban, South Africa). Ferritin was measured using the electrochemiluminescence immunoassay (Modular Analytics E170, Roche Diagnostics, Switzerland. Soluble transferrin receptor (sTfR) levels were measured using ELISA (Ramco Laboratories, TX, USA). Iron deficiency was defined as a sTfR/log ferritin index of 5.6 or more [9], [10]. Malaria was defined as the presence of *Plasmodium falciparum* asexual parasites in the blood. Stool samples were examined for helminth infection by Kato-Katz and Polymerase Chain Reaction (PCR) [11]. Urine specimens were examined for *Schistosoma haematobium* using a semiquantative concentration method [12]. Bacterial cultures were carried out only on *cases* and *HC* according to a standard method using an automated BacT/Alert system (BioMérieux Industry, MO, USA) and cultured for 7 and 56 days for routine pathogens and mycobacteria respectively. Mixed growth or growth of Micrococci, Bacillus species or coagulase-negative staphylococci were considered contaminants. HIV testing was performed according to WHO guidelines using two rapid tests (Determine, Abbott-Laboratories, Japan; Unigold, Trinity-Biotech, Ireland). Discordant results and reactive results in children less than 18 months were resolved by PCR [13]. For genetic analysis, DNA was extracted using a Nucleon extraction kit (Amersham biosciences, UK) and tested by Cytronomics and arms-PCR for a predefined set of polymorphisms including glucose-6-phosphate dehydrogenase deficiency (G6PD, variant A- 202/376) [14], sickle cell disease (homozygosity for hemoglobin S) and single nucleotide polymorphism in the promoter region of the genes encoding interleukin-10 (*IL10*) (–1117, −3585, and +4949) [15] and tumor necrosis factor (*TNF*) (−238, −308, and −1031) [16] .

### Statistical analysis

Data were analyzed using SPSS for Windows© version 12 and STATA© version 9. To compare the characteristics of the three groups of children, discrete data were analyzed by the Fisher exact test and continuous data by independent samples Student's t-tests. Survival times were recorded as the duration of follow-up from the date of recruitment or the date of discharge (if hospitalised at time of recruitment) until the date of a severe anaemia episode or post-discharge mortality. Severe anaemia was defined as [Hb] less than 5g/dl during the follow-up period and at least four weeks after a previous severe anaemia episode. Kaplan-Meier curves were plotted for the three groups of children and compared using the Log-rank test.

We investigated the associations between risk factors determined at recruitment and post-discharge all cause mortality during the 18 month follow-up period among *cases*. It was not possible to perform a similar analysis for *HC* and *CC* owing to the few deaths observed in these study groups. Among *cases*, we examined the association with all cause mortality of the following risk factors: residing in an urban/rural area, sex (male/female), age (in months), education of mothers of study participants (none/some), employment of parents (unemployed/employed), number of siblings (>2 siblings/≤2 siblings), history of previous blood transfusion (yes/no), history of jaundice (yes/no), blood in urine or stool (present/absent), wasting (<−2 Z-score weight-for-height/≥−2 Z-score weight-for-height), stunting (<−2 Z-score height-for-age/≥−2 Z-score height-for-age), undernutrition (<−2 Z-score weight-for-age/≥−2 Z-score weight-for-age), splenomegaly (present/absent), CRP (≤10 mmol/L/>10mmol/L), iron deficiency (≥5.6 sTfR/Log ferritin/<5.6 sTfR/Log ferritin), hookworm (present/absent), malaria (0/any asexual parasites/µL blood), HIV infection (positive/negative), bacteraemia (present/absent), non-typhoidal salmonella bacteraemia (present/absent).

Cox proportional hazards models were used to examine the association between risk factors mentioned above and all cause mortality through the 18 months post-discharge follow-up period. Initially, each covariate (risk factor) was entered into a separate univariate model with time to post-discharge death entered as the dependent variable. All covariates with p≤0.10 in the univariate models and important confounders (age, sex, residency and HIV status) were entered into a multivariable model. We also compared the proportion of children with HIV infection by study group using chi-squared test and determined the respective risk ratios.

We performed a similar analysis (as that for all cause mortality) to assess risk factors for the development of first recurrence of severe anaemia during the follow-up period among *cases*. This analysis was not possible for *HC* and *CC* owing to the few recurrences of severe anaemia in these study groups.

## Results

A total of 381 cases, 377 HC children and 380 CC children were recruited; 4 cases had to be excluded from the statistical analysis because outcome information was missing, reducing this cohort to 377 children. Of all children, 53.4% (606/1134) were from the urban site (Blantyre). Over the 18 months study period 17.8% (63/353) of cases, 19% (74/377) of HC and 15.3% (58/380) of CC were lost to follow-up, and the commonest reasons being emigration out of the study area and withdrawal of consent. There were no significant differences in baseline characteristics of the children lost to follow-up compared to those that completed the study follow-up period.

There were a few differences in the baseline characteristics of the three groups (Table 1). *Cases* had a higher prevalence of stunting, wasting and malaria compared to controls. It was found that iron deficiency, determined in a subgroup of children only, was less common in cases (46.4%, 96/207) than in *HC* (65.7%, 136/207, p<0.001 ) or *CC* (73.1%, 152/208, p<0.001). Bacteraemia was more common among *cases* (15.2%, 54/355) than *HC* (4.0%, 14/353) with 72.1% (49/68) caused by non-typhoidal Salmonella. Blood cultures were not done on *CC*. HIV prevalence was significantly higher among the *cases* (12.7%, 45/553) than *HC* (8.1%, 27/335, p = 0.04) or *CC* (4.0%, 14/347, p<0.001).

**Table 1:** Baseline characteristics by study group.

Baseline characteristics	Cases	Hospital Controls	Community Controls
Urban site	202/377 (53.6%)	201/377 (53.3%)	203/380 (53.4%)
Age (in months)^1^	20.4 (12.8)	22.5 (12.1)	25.3 (13.1)
Male	175/377 (46.4%)	197/377 (52.3%)	189/380 (49.7%)
History of previous transfusion	57/376 (15.2%)	22/376 (5.9%)	16/380 (4.2%)
Educated mother	321/364 (88.2%)	258/377 (68.4%)	296/376 (78.7%)
Wasting (z-score ≤2)	52/328 (15.9%)	28/339 (8.3%)	15/356 (4.2%)
Stunting (z-score ≤2)	175/329 (53.2%)	125/340 (36.8%)	162/357 (45.4%)
[Hb]^2^ (g/dl)^1^	3.6 (0.8)	9.6 (2.2)	9.9 (1.9)
Iron deficiency	96/207 (46.4%)	136/207 (65.7%)	152/208 (73.1%)
Malaria	222/376 (59.0%)	154/376 (41.0%)	167/374 (44.7%)
HIV	45/353 (12.7%)	27/335 (8.1%)	14/347 (4.0%)
Bacteraemia^3^	54/355 (15.2%)	14/353 (4.0%)	–

1 mean (sd);

2 Haemoglobin;

3 blood cultures were not done on community controls

### Incidence of death in study groups

**Figure 1:**
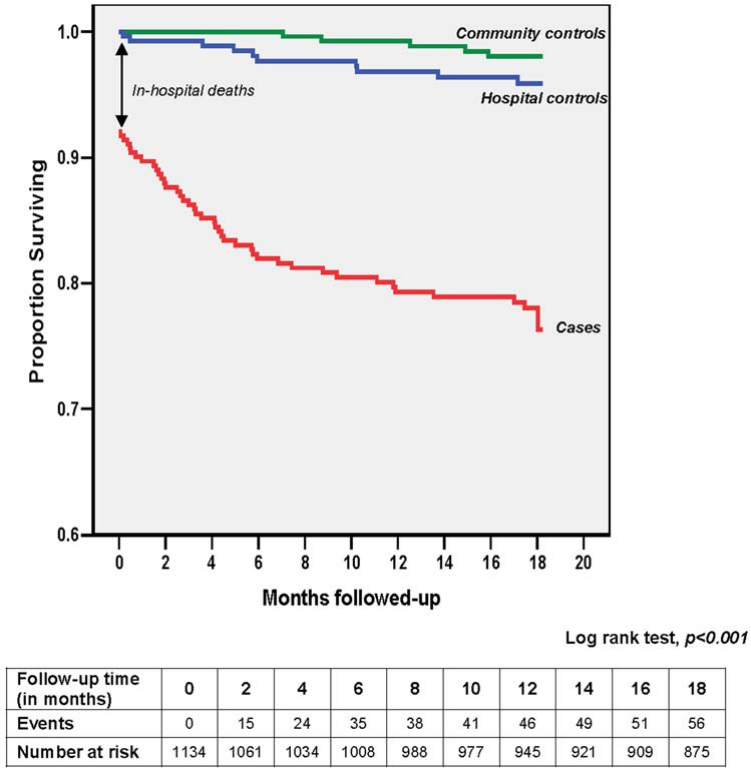
Survival curve showing the time to post-discharge death and the events versus number at risk of severely anaemic children (*cases*) and hospital and community controls during the follow-up period.

The overall all cause mortality rate (in-hospital and post-discharge) was significantly higher among the *cases*, 17.2% (65/377), than the combined control groups 2.0% (15/757, p<0.001, Table 2). The incidence of death among c*ases* was 0.148 per child-year (95% CI 0.116–0.189) and they were ten times more likely to die than controls (HR 9.5, 95% CI 5.4–16.7). Of these deaths among the *cases*, 36.9% (24/65) occurred in hospital (mean hospital stay in days = 4.1±5.0SD) while the remaining 63.1% (41/65) occurred during the post-discharge18 month follow-up period. Most post-discharge deaths occurred in the community and hence their cause could not be exactly determined, however most deaths followed after a short history of illness. There were no deaths that could have been remotely related to anaemia e.g. trauma. The Kaplan-Meier plot (using censored data) of the three cohorts of children (Figure 1) shows a significantly lower proportion of *cases* than *HC or CC* surviving until the end of the follow-up period. The post-discharge all cause mortality among the *cases* was 12.6% compared to 2.9% and 1.4% in the HC and CC groups respectively (Log rank test, p<0.001). Crude post-discharge death rates not using censored data are presented in Table 2. Post-discharge deaths among *cases* occurred after a median time of 4.1 months (IQR 1.8–8.1), with 70.7% (29) occurring within the first six months.

**Table 2:** All cause mortality and morbidity by study group.

	Cases	Hospital Controls	Community Controls
**Follow-up**			
Post-discharge follow-up (days)^2^	537 (0–715)	540 (0–700)	540 (0–694)
Observation time (child-year)	440.1	506.1	535.0
Losses to follow-up at:			
6 months	24/353 (6.8%)	23/377 (6.1%)	14/380 (3.7%)
12 months	36/353 (10.2%)	40/377 (10.6%)	26/380 (6.8%)
18 months	63/353 (17.8%)	74/377 (19.6%)	58/380 (15.3%)
**Mortality**			
Total deaths	65	10	5
All cause mortality incidence^1^	0.148 (0.116–0.189)	0.020 (0.011–0.037)	0.009 (0.003–0.022)
In-hospital mortality	24/377 (6.4%)	0/377 (0%)	–
Total deaths post-discharge	41	10	5
Crude post-discharge mortality^3^ at:			
6 months	29/353 (8.2%)	6/377 (1.6%)	0/380 (0%)
12 months	36/353 (10.2%)	8/377 (2.1%)	2/380 (0.5%)
18 months	41/353 (11.6%)	10/377 (2.7%)	5/380 (1.3%)
**Severe anaemia post-discharge** 4			
Number of children (episodes)	32 (42)	4 (5)	2 (2)
Incidence of severe anaemia^1,5^	0.102 (0.075–0.138)	0.010 (0.004–0.024)	0.004 (0.001–0.016)
One or more episodes^6^ at:			
6 months	21/353 (5.9%)	2/377 (0.5%)	1/380 (0.3%)
12 months	29/353 (8.2%)	4/377 (1.1%)	2/380 (0.5%)
18 months	32/353 (9.1%)	4/377 (1.1%)	2/380 (0.5%)
**Other morbidity**			
Number of sick-clinic visits^7^	910	1185	791
Malaria positive^8^ sick-clinic visits	384/871 (44.1%)	382/1131 (33.8%)	339/761 (44.5%)
Re-admissions to hospital^9^	64/373 (17.2%)	35/371 (9.4%)	34/341 (10.0%)
Frequency of re-admissions:			
None	309	337	341
1 time	45	29	32
2 times	14	4	2
3 times	5	2	0

1 per child-year (95% Confidence Interval);

2 median (range);

3 assuming that all children loss to follow-up survived;

4 all severe anaemia episodes were admitted to hospital and transfused;

5 incidence based on number of episodes of severe anaemia;

6 assuming that all children lost to follow-up did not have a severe anaemia episode;

7 presented to study clinic due to illness;

8 any asexual parasites/µL blood;

9 number of children with at least one re-admission to hospital

### Risk factors for death in children treated for severe anaemia (*cases*)

Nearly half the children who died, 45% (27/60) were infected with HIV (Table 3). Among all study groups of children, HIV infection was associated with an increased risk of dying (*cases* HR 9.4, 95% CI 5.0–17.5*; HC* HR 12.7, 95% CI 3.2–50.8*; CC* HR 17.1, 95% CI 2.9–102.2).

**Table 3:** Baseline characteristics of severe anaemia cases by outcome.

	All deaths (n = 65)	Post-discharge mortality (n = 41)	Survivors (n = 312)
Urban site	41/65 (63.1%)	25/41 (61.0 %)	161/312 (51.6%)
Age (in months)^1^	19.6 (12.5)	17.8 (10.8)	20.5 (12.8)
Male	25/65 (38.5%)	19/41 (46.3%)	150/312 (48.1%)
History of previous transfusion	9/64 (14.1%)	5/41 (12.2%)	48/312 (15.4%)
Educated mother	50/58 (86.2%)	31/38 (81.6%)	271/306 (88.6%)
Wasting	15/51 (29.4%)	7/35 (20.0%)	37/277 (13.4%)
Stunting	32/51 (62.7%)	23/35 (65.7%)	143/278 (51.4%)
Splenomegaly	37/63 (58.7%)	20/40 (50.0%)	198/307 (64.5%)
Hb (g/dl)^1^	3.4 (0.8)	3.5 (0.6)	3.6 (0.9)
Iron deficiency	16/32 (50.0%)	13/25 (52.0%)	80/175 (45.7%)
Malaria	30/64 (46.9%)	22/41 (53.7%)	192/312 (61.5%)
HIV	27/60 (45.0%)	20/41 (48.8%)	18/293 (5.8%)^3^
Bacteraemia	18/57 (31.6%)	12/41 (29.3%)	36/298 (12.1%)

1 mean (SD);

3 *p<0.0001* between Survivors and All deaths or post-discharge mortality

**Figure 2:**
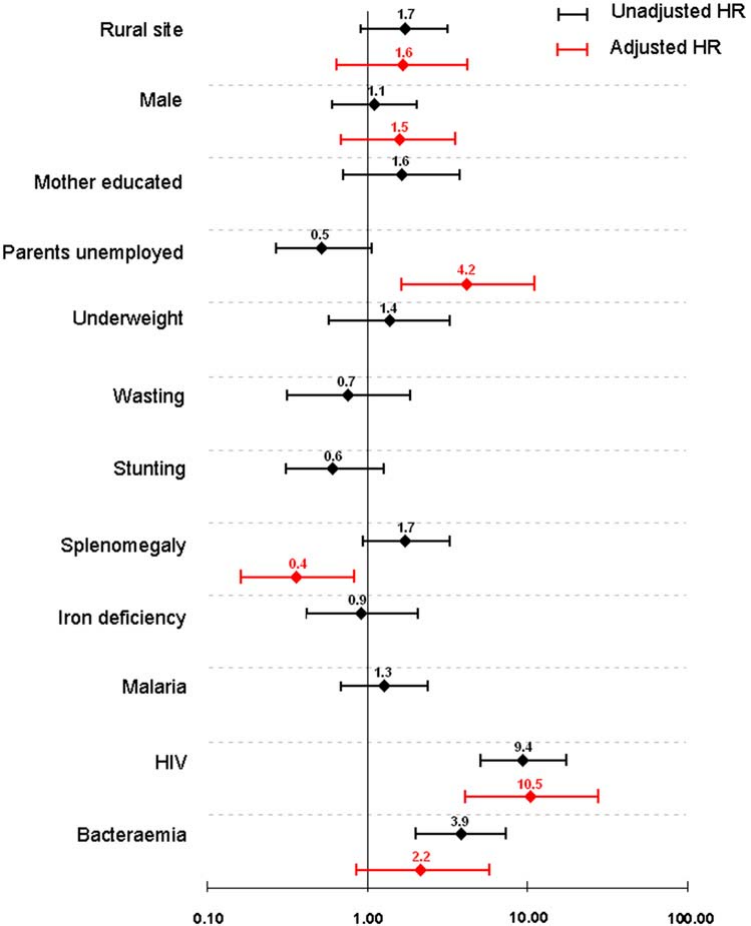
Unadjusted (in black) and adjusted (in red) Hazard Ratios of main risk factors for post-discharge all cause mortality among severely anaemic children (*cases*).

For *cases*, risk factors for post-discharge all cause mortality are presented in Figure 2 and Table 4. Children who died were more commonly HIV-infected than survivors and after adjusting for confounders, HIV was strongly associated with death in the follow-up period (HR 10.5, 95% CI 4.0–27.2). Overall all cause mortality was 60.0% (27/45) among HIV infected and 10.7% (33/308) among HIV uninfected severely anaemic children (p<0.001). Bacteraemia was associated with a non-significantly increased risk of post-discharge all cause mortality (HR 2.2, 95% CI 0.8–5.6). Malaria, study site, sickle cell disease, G6PD or hookworm infection did not significantly predict post-discharge death. Each unit increase in age (in months) was associated with HR 0.92 (95% CI 0.87–0.97, p = 0.001).

**Table 4:** Risk factors for post-discharge all cause mortality among cases.

Factor	No. of children^1^	Univariate HR^2^ (95% CI^3^)	p-value	Adjusted HR (95% CI)^4^	p-value
**Age (in months)**					
Unit increase in age^5^	345	0.98 (0.95–1.01)	0.2	0.92 (0.87–0.97)	0.001
**Residency**					
Rural	175	1.72 (0.90–3.23)	0.10	1.63 (0.63–4.20)	0.31
Urban	202	1.00			
**Sex**					
Male	175	1.09 (0.58–2.02)	0.80	1.54 (0.68–3.52)	0.31
Female	202	1.00			
**Mother education**					
Some	321	1.63 (0.72–3.70)	0.25	NA^6^	
none	43	1.00			
**Parents employment**					
Unemployed	214	0.51 (0.26–1.04)	0.06	4.15 (1.61–10.74)	0.003
Employed	158	1.00			
**Wasting**					
<−2 Z-score WH^7^	52	0.74 (0.31–1.80)	0.51	NA	
≥−2 Z score WH	276	1.00			
**Stunting**					
<−2 Z-score HA^8^	175	0.61 (0.30–1.22)	0.16	NA	
≥−2 Z score HA	154	1.00			
**Splenomegaly**					
Present	235	1.73 (0.92–3.23)	0.09	0.36 (0.16–0.80)	0.01
Absent	135	1.00			
**Iron deficiency**					
≥5.6 sTfR/Log ferritin	96	0.91 (0.41–2.03)	0.82	NA	
<5.6 sTfR/Log ferritin	111	1.00			
**Malaria**					
Any parasite/µL blood	222	1.25 (0.67–2.34)	0.48	NA	
0 parasite/µL blood	154	1.00			
**HIV**					
Positive	45	9.39 (5.05–17.49)	<0.0001	10.49 (4.05–27.20)	<0.0001
Negative	308	1.00			
**Bacteraemia**					
Present	54	3.85 (2.00–7.14)	0.001	2.17 (0.84–5.64)	0.11
Absent	301	1.00			

1 number of children included in Univariate model;

2 Hazard Ratio;

3 Confidence Interval;

4 number of children included in the multivariable Cox Proportional Hazards model was 272. All variables with a univariate HR p-value ≤0.1 and important confounders (age, sex, residency, HIV) were included in the multivariable model. Other factors not presented in the table that were included in the model were history of a previous transfusion, Hb at admission;

5 age was entered as a continuous variable in the model;

6 not applicable;

7 weight-for-height;

8 height-for-age

### Incidence of severe anaemia in study groups

**Figure 3:**
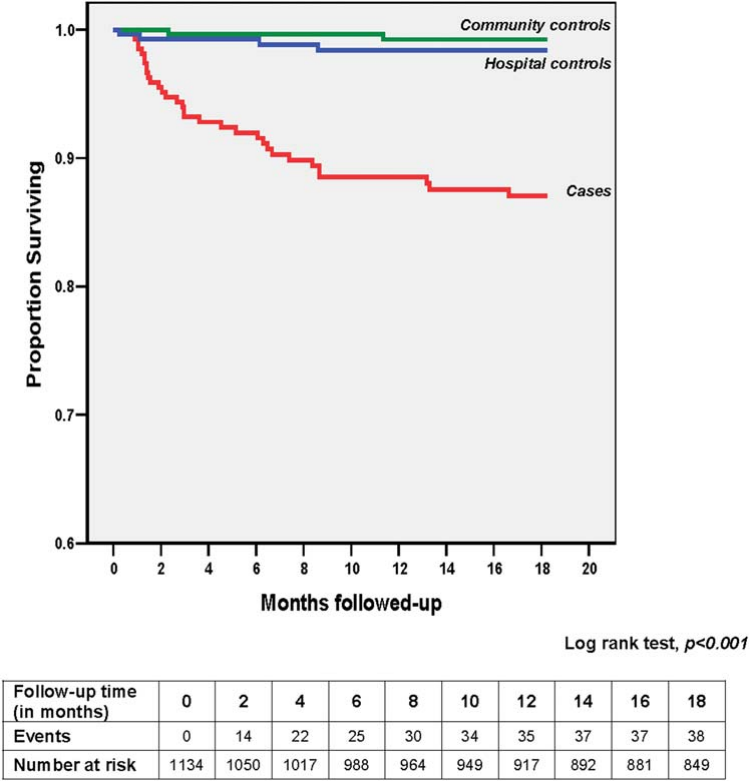
Survival curve showing the time to first severe anaemia episode and the events versus number at risk of severely anaemic children (*cases*) and hospital and community controls during the follow-up period.

Recurrence of first episode of severe anaemia was observed in 10.2% (Kaplan-Meier estimates) of *cases* during the 18-month follow-up period, with a median time to event of 2.9 months (IQR 1.4–6.6) (Figure 3). *Cases* had a significantly higher rate of severe anaemic episodes (Log rank test, p<0.001) during the follow-up period than *HC* (1.1%) or *CC* (0.6%). Crude estimates of rates of recurrence of first episode of severe anaemia are presented in Table 2. Of these children 65.6% (21/32) had their first episode occurring within the first six months after discharge from hospital. The incidence of severe anaemia in the follow-up period among the *cases* was 0.102 per person-year (95% CI 0.075–0.138) compared to 0.007 per child-year (95% CI 0.003–0.015) for the combined controls (p<0.0001). All children with an episode of severe anaemia during the follow-up period were re-admitted to hospital and given a blood transfusion.

### Risk factors for recurrence of severe anaemia in children treated for severe anaemia (*cases*)

Having more than 2 siblings, was strongly associated with an increased risk of severe anaemia (AHR 5.0 95% CI 1.9–13.3) (Table 5). From the history on admission, *cases* who had had a previous transfusion or symptom of jaundice were more likely to develop severe anaemia during the follow-up period (AHR 2.9, 95% CI 1.0–8.9; and AHR 11.8 95% CI 3.9–36.0 respectively). Malaria on admission was associated with a protective effect, with malaria affected *cases* having an 80% reduced risk of recurrence of severe anaemia (AHR 0.2, 95% CI 0.1–0.7). HIV infection was associated with a five times increased risk of recurrent severe anaemia (AHR 5.6, 95% CI 1.6–20.1) among *cases*. Overall, among all study groups of children, univariate analysis showed that HIV infection was non-significantly associated with severe anaemia (*cases* HR 1.9, 95% CI 0.7–5.0*; HC* HR 4.2, 95% CI 0.4–40.6*; CC*-too few events to compute HR).

**Table 5:** Risk factors for recurrence of severe anaemia among cases.

Factor	No. of children^1^	Univariate HR^2^ (95% CI^3^)	p-value	Adjusted HR (95% CI)^4^	p-value
**Age (in months)**					
Unit increase in age^5^	340	1.00 (0.97–1.03)	0.9	0.97 (0.94–1.00)	0.08
**Residency**					
Rural	175	0.76 (0.38–1.52)	0.44	0.63 (0.25–1.60)	0.33
Urban	202	1.00			
**Sex**					
Male	175	0.57 (0.27–1.17)	0.13	0.61 (0.23–1.61)	0.31
Female	202	1.00			
**No. of siblings**					
>2	171	2.09 (1.00–4.37)	0.05	4.98 (1.87–13.25)	0.001
≤2	204	1.00			
**History of previous transfusion**					
Yes	57	1.65 (0.71–3.81)	0.24	NA^6^	
No	319	1.00			
**History of jaundice**					
Yes	22	4.23 (1.74–10.32)	0.002	11.84 (3.90–35.96)	<0.0001
No	350	1.00			
**Splenomegaly**					
Present	235	0.41 (0.21–0.83)	0.01	0.98 (0.38–2.51)	0.98
Absent	135	1.00			
**Iron deficiency**					
≥5.6 sTfR/Log ferritin	96	0.98 (0.40–2.42)	0.97	NA	
<5.6 sTfR/Log ferritin	111	1.00			
**Malaria**					
Any parasite/µL blood	222	0.38 (0.18–0.77)	0.007	0.24 (0.09–0.65)	0.005
0 parasite/µL blood	154	1.00			
**HIV**					
Positive	45	1.91 (0.73–4.99)	0.19	5.63 (1.58–20.07)	0.008
Negative	308	1.00			
**Bacteraemia**					
Present	54	1.05 (0.37–3.00)	0.93	NA	
Absent	301	1.00			

1 number of children included in Univariate model;

2 Hazard Ratio;

3 Confidence Interval;

4 number of children included in the multivariable Cox Proportional Hazards model was 307. All variables with a univariate HR p-value ≤0.1 and important confounders (age, sex, residency, HIV) were included in the multivariable model. The other factor not presented in the table but included in the model was undernutrition;

5 age was entered as a continuous variable in the model;

6 not applicable

### Other morbidity during the follow-up period

There were 910, 1185 and 791 sick-clinic visits for cases *HC* and *CC* respectively (Table 2). Of these visits, there was a significantly reduced proportion of malaria positive sick-visits among the *HC* than *cases* or *CC*. (382/1131 *HC* vs. 384/871 *cases*, p = 0.002; *HC* vs. 339/761 *CC*, p = 0.002). During the follow-up period, a higher proportion of *cases* were re-admitted to hospital (64/373, 17.2%) as compared to *HC* (35/371, 9.4%, p = 0.007) and *CC* (34/341, 10.0%, p = 0.01). These proportions were not significantly different between the *HC* and *CC* (p = 0.8).

## Discussion

The in-hospital case-fatality rate of 6.4% in the present study is within the range of previously reported studies in Sub-Saharan Africa [2], [3], [17]. In-hospital deaths usually occur shortly after admission and respiratory distress has been found to be an important predictor of a fatal outcome [3]. Earlier identification of these children in the community and more timely transfusion may decrease these early deaths.

Of serious concern is the high post-discharge all cause mortality observed among the cases in this study, most deaths occurring within six months after admission for severe anaemia. The all cause mortality rate over the same period was significantly lower in other children attending the hospital (hospital controls) and in children in the community (community controls). Children with severe anaemia, even if treated in hospital, remain a highly vulnerable group after discharge. Outside of the context of a research study, such deaths may remain undetected by the health services, and if recorded, the link with a previous episode of severe anaemia may be unrecognized.

Before this study the longest follow-up of children after an episode of severe anaemia was eight weeks, in an investigation in rural Kenya [2]. Even in that relatively short period, the post-discharge all cause mortality rate was found to be 14%, which the investigators attributed to suboptimal medical care during admission to hospital (poor disease recognition and inadequate treatment). The present study has been the first attempt to validate these Kenyan findings in a different setting and with an extended follow-up period. We provided a good level of supervised medical care during the initial admission, and were therefore surprised to find that both post-discharge all cause mortality and the rate of recurrence of severe anaemia, although better than in Kenya, remained high.

Several possible explanations for the high incidence of post-discharge all cause mortality and recurrent severe anaemia should be considered. Firstly, it is possible that the underlying cause of the initial episode of severe anaemia was neither diagnosed nor treated, and therefore haemoglobin levels may have deteriorated again after discharge. Considering the extensive diagnostic investigations [6] and frequent follow-up visits that were carried out by dedicated medical staff, this explanation appears unlikely.

Secondly, the aetiology of the severe anaemia episode may have been identified correctly, but treatment failure occurred as a result of multi-drug resistant malaria or bacterial infections. It has been shown that recrudescent and new malaria infections during the first six months can negate the initial haematological improvement attained from receiving blood transfusion [2]. Children with malaria in this study were treated with a combination of quinine for at least three days and sulfadoxine-pyrimethamine according to national policy. Although generally efficacious, this regimen is known to have an increasing failure rate in formal studies in Malawi, so that recrudescent malaria may have contributed to recurrent anaemia in some children. However if failed malaria treatment had been an important explanation of recurrent anaemia, we might have expected recurrence to occur more frequently among cases who had malaria parasitaemia at the time of admission; in fact these children proved to have a lower incidence of recurrent anaemia than those without malaria.

Thirdly, *cases* may have been seen in the end-stage of a chronic underlying disease, with severe anaemia being merely a marker of disease severity. This hypothesis is supported by the fact that HIV infection was found to be the most important independent risk factor for post-discharge all cause mortality. Anaemia is not only a common paediatric manifestation of HIV, but has been shown to be strongly and consistently associated with the progression of HIV disease and death [18], [19]. At the time of this study, anti-retroviral therapy (ART) was not available to children in Malawi. Currently ‘unexplained moderate anaemia’ is a stage III criterion–i.e. a provisional indication–for commencing ART, but in practice this is often not used in deciding which children should be started on ART. Our finding of a very high HIV-related case fatality rate in severely anaemic children suggests that it is now appropriate to consider adding severe anaemia to the criteria for stage IV disease–ie a definite indication for commencing ART–in the WHO paediatric HIV staging system.

Finally, inadequate blood transfusion may explain the high rates of post-discharge all cause mortality and recurrent severe anaemia, an explanation also suggested in the Kenyan study [2]. We have tried to prevent this happening by evaluating haemoglobin levels after transfusion and before discharge, and repeating transfusions if necessary. Nevertheless, in *cases* haemoglobin concentrations were still well below the normal range following transfusion–a situation that is likely to prevail in many health facilities in Africa–and a relatively minor additional haematological insult could have major consequences.

Iron status determination in populations were inflammatory conditions are common can be challenging. In this study sTfR-Log ferritin index was used after assessment with bone marrow iron aspirates found it to best predict bone marrow iron status [10]. However, other studies where sTfR and ferritin were assessed separately, these iron status markers showed large alterations with inflammation [20], [21], [22]. Although iron deficiency was not associated with an increased risk of post-discharge all cause mortality, it was less prevalent among the cases on admission than among controls. This observation supports the hypothesis that iron deficiency may provide an unfavourable environment for bacterial growth [23]. It is also in agreement with observations of increased morbidity and mortality during iron-supplementation studies in areas where bacterial infections are common [24]. However the effect of iron supplementation in this present study could not be properly determined.

The importance of bacteraemia has been shown in a previous study in Malawi, where it was associated with severe anaemia [25]. Non-typhoidal salmonellae, principally *S. typhimurium* and *S. enteritidis*, are widely prevalent in Africa and are associated with malaria and HIV infection [26]. In our study bacteraemia was more common among *cases* than *HC*, though a comparison with *CC* could not be made. This association may be due in part to the higher prevalence of malaria and HIV infection in cases than controls, malaria and HIV being known risk factors for bacteraemia. Bacteraemia was not an independent risk factor for post discharge all cause mortality or recurrence of severe anaemia. Whether routine treatment with antibiotics should be given to all children with severe anaemia remains a matter of debate. Our data suggest that this would be a reasonable policy where blood cultures cannot be carried out.

Our analysis included only risk factors that could be ascertained at the time of admission. Causes of a post-discharge death, if it occurred in the community, could only be investigated retrospectively by using the verbal autopsy method. Verbal autopsies are known to be neither very sensitive nor specific; therefore the true causes of post-discharge all cause mortality in the study population could not be clearly determined [27]. A limitation to the study was that it was carried out during the time when ART was not freely available to children. The impact of HIV infection on post-discharge all cause mortality in severely anaemic children is likely to be less in children to whom ART is available.

This study provides disturbing evidence of consequences of severe anaemia on child health and survival. It is commonly thought that most deaths due to severe anaemia occur in hospital [3]. This study shows that there is an even greater mortality post-discharge. This ‘hidden’ post-discharge all cause mortality, calculated at 12.6% during 18 months (compared to 2% in the combined controls), is likely to be an underestimate, as it is based on the assumption that all children lost to follow-up survived. We have also shown that severe anaemia is followed by an increased risk of further episodes of severe anaemia. In this study, HIV infection proved to be the most important risk factor both for recurrent anaemia and for death during the follow-up period. As severe anaemia is very common [2], the impact on overall under-five morbidity and mortality is likely to be considerable. Increased attention to the prevention and management of underlying diseases causing severe anaemia in African children is urgently needed if the fourth millennium development goal (MDG4)–a significant reduction in child mortality–is to be achieved.
